# Pseudotumoral tuberculous ureteritis: a case report

**DOI:** 10.1186/1752-1947-7-45

**Published:** 2013-02-15

**Authors:** Ahmed-Amine Bouchikhi, Driss Amiroune, Mohammed Fadl Tazi, Soufiane Mellas, Jalal Eddine Elammari, Mohammed Jamal El Fassi, Abdelhak Khallouk, Moulay Hassan Farih

**Affiliations:** 1 Urology Department, University Hospital of Fez, Rue Zag, Résidence Andalous III, 30070, Quartier Al-Wafae Fès, Morocco

**Keywords:** Pseudotumor, Tuberculosis, Ureter

## Abstract

**Introduction:**

Tuberculosis is still endemic in Morocco and the urogenital form is common. This form is characterized by clinical polymorphism. However, the isolated ureteric form is very rare. The differential diagnosis might be raised in tumoral cases while undertaking surgical excision which is the realistic choice. Hence, we report an isolated ureteric tuberculosis case, and we discuss the clinical, imaging, diagnostic and therapeutical features.

**Case presentation:**

A 30-year-old Moroccan man consulted us for left back pain associated with urinary frequency and a few macroscopic episodes of hematuria for the past six months. A computed tomography urography revealed a left hydronephrosis and hydroureter secondary to focal wall thickening of the left lumbar ureter. Hence, we had diagnosed a ureteral tumor. However, a clinical examination showed irritative voiding symptoms and epididymal disorders associated with prostate infection suggesting a Koch’s bacillus assessment of the patient’s urine of which the results proved strongly positive. The treatment consisted of establishing a double-J ureteric stent to drain the left kidney, followed by antituberculous antibiotics.

**Conclusion:**

Urogenital tuberculosis is common in endemic countries, however isolated ureter affection is rare. It is important to consider a ureteral tuberculosis diagnosis whenever ureteral thickening is revealed in a patient living in a country in which tuberculosis is endemic.

## Introduction

Urogenital tuberculosis is frequently characterized by clinical polymorphism, but isolated ureteral infection is rare even in countries in which tuberculosis is endemic. The differential diagnosis might be raised in tumoral cases while undertaking surgical excision which is the realistic choice.

We report a case of pseudotumoral tuberculous ureteritis in a 30-year-old man who presented with left back pain associated with voiding irritation syndrome. We discuss the clinical, imaging, diagnostic and therapeutical features.

## Case presentation

Our case was a 30-year-old Moroccan man without significant past medical history. The patient consulted us for left back pain associated with urinary symptoms of urinary frequency, and macroscopic hematuria for the past six months. The physical examination revealed an exhausted patient. The patient presented with anorexia and significant weight loss: his weight had fallen by 10kg in six months. The urogenital examination found a left epididymitis and normal prostate size with slight loss of elastic consistence; his prostate was firmer than usual for a young patient and without nodules.

**Figure 1:**
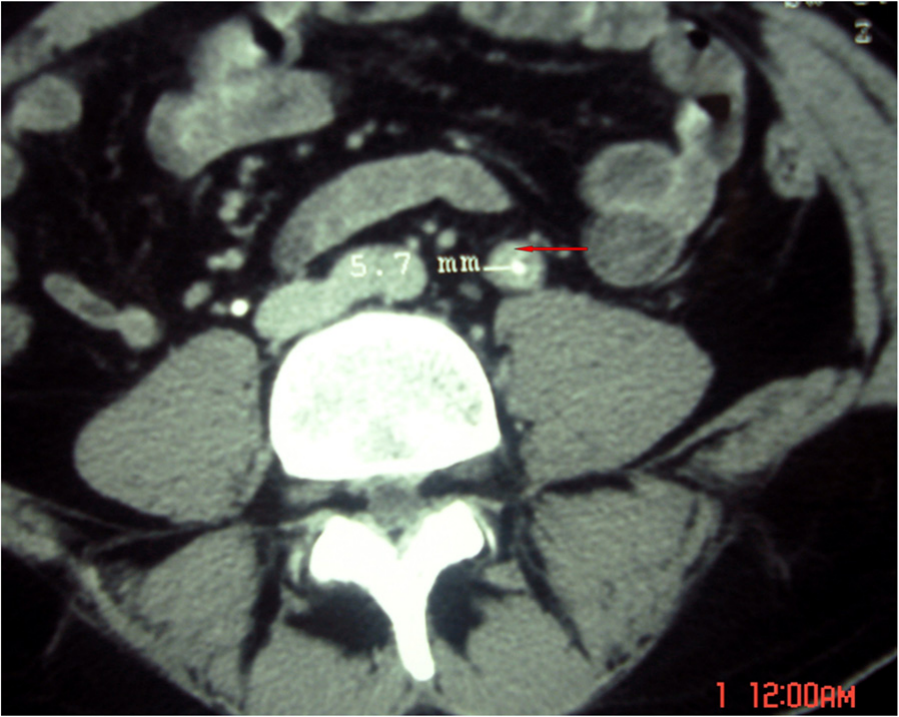
The computed tomography scan showed thickening of the tumor aspect in the left lumbar ureter (red arrow) of tuberculous origin.

Laboratory investigations were performed and showed appropriate kidney function with a creatinine of 66.2μmol/L, an inflammatory syndrome with an erythrocyte sedimentation rate of 120mm/hour and a C-reactive protein of 200mg/L, whereas the remaining laboratory investigations were unremarkable. The patient then underwent a renal and pelvic ultrasound which showed left hydronephrosis and hydroureter. This examination was completed by a computed tomography (CT) urography that revealed a left ureterohydronephrosis in the left lumbar ureter secondary to focal wall thickening (Figure 1). Thus, it was thought that we revealed a ureteral tumor. However, considering the irritative voiding symptoms, epididymal symptoms, and slight indurations of the prostate, we suggested a Koch’s bacillus assessment of the patient’s urine of which the results proved strongly positive.

The treatment consisted of establishing a ureteric double-J stent to drain the left kidney, followed by antituberculous antibiotics.

## Discussion

Urogenital tuberculosis is characterized by varied clinical symptoms and a lack of specific clinical signs [1]. Ureteral localization was always described as secondary to renal disease because it represents the first ureteral mucosal lesions extending from the kidney lesions [2,3]. The case reported here did not reveal any visible renal impairment by imaging exploration except the dilatation of pyelocaliceal cavities. Pseudotumoral ureter tuberculosis is rare [4,5]. It is probably due to an extending fibro-inflammatory process in the thickening of the ureteral wall; this could be confused with a ureteral tumor in imaging results.

A clinical examination is crucial for an accurate diagnosis. Indeed, it allowed the retrieval of the patient’s history of tuberculosis and tuberculosis contagion signs; a clinical examination of the patient allows orientation towards urogenital tuberculosis with epididymo-testicular damage, prostate and kidney involvement.

The diagnosis confirmation is based on assessing Koch’s bacilli in the urine by direct testing for alcohol-acid-resistant bacillus. The Koch’s bacillus culture has to be achieved considering a strong suspicion of Koch’s bacillus with a negative direct examination or antibiotic susceptibility testing (Koch’s bacillus sensitivity to antibacillary agents). However, the delay for obtaining the final results is long and might last eight weeks.

The technology for identifying Koch’s bacillus using polymerase chain reaction is faster and takes 24 to 48 hours, but with a sensitivity reduced to 48.5%.

An anatomopathological examination helps to confirm the tuberculosis diagnosis because it shows a breach of epithelial giant cells with the presence of caseous necrosis.

The CT urography and intravenous urography with micturition examinations are designed to make an extended assessment of the urogenital tuberculosis lesions. These approaches allow studying the kidney and urinary meatus.

The treatment consists of antibacillary medication and surgical urinary drainage in case of urinary tract obstruction. This includes the implementation of a double-J ureteric stent and percutaneous nephrostomy in the initial stage. Reconstructive or removal endoscopic surgeries are to be considered depending on the degree and nature of injury consequences such as ureter stenosis, destroyed kidney and small bladder.

## Conclusion

Urogenital tuberculosis is a frequent pathological entity. Isolated ureter affection is rare even in countries in which tuberculosis is endemic. It is important to suggest a ureteral tuberculosis diagnosis whenever ureteral thickening is revealed in a patient living in a country in which tuberculosis is endemic.

## Consent

Written informed consent was obtained from the patient for publication of this case report and any accompanying images. A copy of the written consent is available for review by the Editor-in-Chief of this journal.

## Competing interests

The authors declare that they have no competing interests.

## Authors’ contributions

AAB was the principal author and major contributor in writing the manuscript. MFT, SM, JE, AK and DA analyzed and interpreted the patient data and reviewed the literature. MJE, AK and MHF read and corrected the manuscript. All authors read and approved the final manuscript.
